# Prognostic accuracy of transcranial magnetic stimulation-induced motor evoked potentials on recovery of upper limb: a systematic review

**DOI:** 10.3389/fneur.2026.1788829

**Published:** 2026-05-07

**Authors:** Johanna C. M. Schilder, Maurits H. J. Hoonhorst, Ralph de Vries, Gert Kwakkel

**Affiliations:** 1 Department of Rehabilitation Medicine, Amsterdam UMC location University of Amsterdam, Amsterdam, Netherlands; 2 Department of Neurorehabilitation, Rehabilitation Centre Vogellanden, Zwolle, Netherlands; 3 Medical Library, Amsterdam UMC location Vrije Universiteit Amsterdam, Amsterdam, Netherlands; 4 Department of Neurorehabilitation, Amsterdam Rehabilitation Research Centre Reade, Amsterdam, Netherlands; 5 Department of Physical Therapy and Human Movement Sciences, Northwestern University, Chicago, IL, United States; 6 Department of Rehabilitation Medicine, Amsterdam UMC location Vrije Universiteit Amsterdam, Amsterdam Neurosciences and Amsterdam Movement Sciences, Amsterdam, Netherlands

**Keywords:** corticospinal tract, motor evoked potential, motor recovery, prognostic accuracy, stroke, transcranial magnetic stimulation, upper extremity

## Abstract

**Background:**

Prospective prognostic studies examining transcranial magnetic stimulation-induced motor evoked potentials (TMS-MEP) as predictors of upper limb (UL) motor function and capacity following stroke are influenced by variations in TMS procedures, outcome measures, and the timing of assessments. This prognostic review investigated three key areas: 1) the technical and methodological quality of TMS-MEP studies; 2) the prognostic accuracy for outcome related to muscle strength, muscle synergies and UL-capacity; and 3) the influence of the timing of TMS-MEP on outcomes.

**Methods:**

With the QUAPAS (Quality Assessment of Prognostic Accuracy Studies) tool and Chipchase checklist the risk of bias (RoB) and technical quality of TMS-MEP studies were assessed, respectively. Sensitivity, specificity, negative predicted values (NPV), and positive predicted values (PPV) were analyzed for muscle strength, muscle synergies, and UL-capacity.

**Results:**

Sixteen prospective TMS-MEP studies were included, all of which showed low RoB. Three studies reported all the recommended technical items for TMS testing. After pooling the data, muscle strength showed a sensitivity of 0.76 (95% CI: 0.64–0.85), specificity of 0.96 (95% CI: 0.55–1.00), PPV of 0.96 (95% CI: 0.85–0.99) and NPV of 0.62 (95% CI: 0.43–0.78). For muscle synergies, sensitivity was 0.70 (95% CI: 0.57–0.81), specificity was 0.98 (95% CI: 0.65–1.00), PPV was 0.98 (95% CI: 0.54–1.00) and NPV was 0.69 (95% CI: 0.42–0.87). For UL-capacity, the sensitivity was 0.84 (95% CI: 0.76–0.89), specificity was 0.91 (95% CI: 0.78–0.96), positive predictive value (PPV) was 0.91 (95% CI: 0.79–0.97), and negative predictive value (NPV) was 0.81 (95% CI: 0.65–0.91). No significant differences in accuracy were observed between type of outcome measures (i.e., muscle strength, muscle synergies or UL-capacity) and no differences were found between groups application of TMS-MEP before or beyond the seventh day after stroke onset.

**Conclusion:**

TMS-MEP accurately predicts favorable UL-motor recovery regarding muscle strength, muscle synergy, and UL-capacity. However, its accuracy in identifying individuals who do not regain UL motor function and capacity remains limited early post-stroke.

## Introduction

Understanding how much upper limb (UL) motor function improves early after stroke is paramount for providing patients and their families with accurate information. This knowledge is also essential for setting realistic treatment goals and developing adequate discharge policies early poststroke. For researchers, early prediction of UL-motor function is important for stratifying randomized clinical trials (1) and creating homogeneous prognostic subgroups in prospective cohort studies. Several prospective studies have shown that some degree of intactness of the corticospinal tract (CST) system is an important prognostic marker for the outcomes of UL-capacity (2, 3) and is a well-known predictor of therapy response (4). In clinical practice, bedside measures such as the Shoulder Abduction and Finger Extension (SAFE) score are valuable clinical markers for recovery of UL-capacity post-stroke (5, 6). Also, different non-invasive techniques have been recommended to improve prognostic accuracy in predicting UL recovery in terms of muscle strength, muscle synergies, and capacity. These techniques include Transcranial Magnetic Stimulation (TMS) (7–9), Somatosensory Evoked Potentials (10), Electro Encephalography (11, 12), and neuroimaging techniques such as Diffusion Tensor Imaging or structural Magnetic Resonance Imaging (13), which serve as markers of CST function (3, 14–16). To improve prognostic accuracy, multimodal approaches have been proposed that combine clinical assessments with neurophysiological and neuroimaging markers (16, 17). The most used non-invasive method to reveal CST integrity involves measuring TMS-induced Motor Evoked Potentials (MEP) in the hand or forearm muscles of the most affected side (3, 13). Regrettably, the majority of studies using this technique have methodological flaws. These issues include small sample sizes, the absence of an inception cohort, and the timing of TMS-MEP assessments, which are often conducted at varying, sometimes arbitrary time points, such as upon discharge from inpatient care. Additionally, timing of reliable and valid outcome measures is not always assessed adequately and preferably evaluated beyond the time window of spontaneous motor recovery of first 3 months post-stroke (18).

In recent years, several narratives (19) and systematic (3, 7, 9, 13, 20) reviews have been published, summarizing the prognostic accuracy of TMS-MEP. Among these prognostic reviews, only one (20) evaluated the methodological and technical quality of the included studies, while another review (3) did investigate the pooled prognostic accuracy by performing a quantitative analysis.

The present research synthesis will first assess the technical and methodological quality of published studies that utilize TMS-MEP to predict UL muscle strength, muscle synergies, and UL-capacity following stroke. Subsequently, we will analyze the pooled prognostic accuracy of the included TMS-MEP studies, focusing on their sensitivity, specificity, as well as their positive (PPV) and negative predictive values (NPV) regarding the three different motor outcomes (muscle strength, muscle synergies and UL-capacity) of interest. In a subsequent heterogeneity analysis, we will investigate how timing of TMS-MEP after stroke onset and type of outcome affect found estimates of precision.

## Methods

### Study identification and selection criteria

This review is reported according to the Preferred Reporting Items for Systematic Reviews and Meta-Analyses (PRISMA) (21). We conducted systematic searches in the bibliographic databases PubMed, Embase.com, Ebsco/APA PsycInfo, Ebsco/CINAHL, Web of Science, Wiley/Cochrane Library, and WHO-ICTRP to identify relevant publications. The search covered publications from inception until May 25, 2023, in collaboration with a medical librarian (RdV). We used various terms, along with synonyms and closely related words, as index terms or free-text searches, including: “Transcranial Magnetic Stimulation,” “Motor Evoked Potential,” “Stroke,” and “Cerebrovascular accident.” Additionally, we reviewed the references of the identified studies to find further relevant publications. To exclude duplicates, a medical information specialist utilized EndNote X20.0.1 (Clarivate), employing the systematic Amsterdam Efficient Deduplication (AED) method (22) and the Bramer method (23). Complete search strategies for all databases are available in the Supplementary Material, Section S1. Two authors (JS and MH) independently screened the titles of the identified references and selected relevant studies based on the titles and abstracts using the web-hosted screening tool Rayyan (24) for this process. Subsequently, they independently verified that the selected studies met the inclusion criteria. If a study did not provide conclusive information in the abstract, the full manuscript was retrieved for further evaluation. No disagreements between the two authors occurred. To ensure the quality, reliability, and reproducibility of the included studies, our systematic review was limited to peer-reviewed publications retrieved from established bibliographic databases.

### Inclusion and exclusion criteria for meta-analysis

Studies were included if they met the following inclusion criteria: 1) participants were at least 18 years of age and had an UL-motor deficit (paresis or paralysis) resulting from either an ischemic or hemorrhagic stroke; 2) the follow-up period for measuring motor or functional recovery was at least 12 weeks post-stroke; 3) the study was an original report and published in English; 4) sufficient data could be extracted from the manuscript or obtained upon request from the author to estimate the accuracy of TMS-MEP as a prognostic biomarker; 5) TMS assessments were conducted prospectively to obtain MEPs from target muscles in the affected hand within the first 8 weeks post-stroke. This timeframe was selected because most spontaneous recovery occurs within the first two months following stroke (25, 26). We excluded case reports, retrospective studies, and studies with fewer than 10 participants, as a minimal number of participants is required to calculate associations between TMS-MEP and outcomes (27). If multiple papers were published based on the same or overlapping datasets, we selected the manuscript that included the largest number of participants.

### Assessment of methodological and technical quality

The methodological quality of the included studies was independently assessed by two authors, JS and MH, using the Quality Assessment of Prognostic Accuracy Studies (QUAPAS) tool (28). This tool, which was recently developed, is designed to evaluate observational prognostic studies and integrate elements from the Quality Assessment of Diagnostic Accuracy Studies 2 (QUADAS-2) (29), the Quality in Prognosis Studies tool (QUIPS) (30), and the Prediction model Risk of Bias Assessment Tool (PROBAST) (31). QUAPAS is specifically recommended for assessing the risk of bias (RoB) regarding the methodological quality of prognostic accuracy studies (32). The QUAPAS tool consists of 18 items that are categorized into five domains: 1) the type of participants recruited for the study; 2) the testing characteristics of the investigated independent factor (e.g., TMS-MEP testing); 3) the psychometric properties of the outcome measurement used; 4) the time from TMS-MEP assessment to the outcome, along with considerations regarding participant lost to follow-up and exclusion from the analysis; and 5) the statistical analysis employed. Additionally, QUAPAS addresses concerns about applicability, which is defined as the extent to which the study findings align with clinical practice (29, 33), as outlined in our research questions. A detailed breakdown of RoB judgments (low, high, or unclear) across all domains is presented in the Supplementary Material, Section S2.

The technical quality of the included studies was assessed using a revised version of the Chipchase checklist (34), which was specifically designed for the transparent evaluation and reporting of items and procedures related to TMS-MEP testing. This checklist was published first in 2012 and contained of 31 items outlining the recommended technical procedures for applying a single-pulse diagnostic TMS-MEP to test intactness of corticospinal motor pathways (34). For this review, we focused exclusively on items that are relevant to prognostic, single-pulse, TMS-MEP studies addressing our research questions (See section 3 of the Supplementary Material). Any disagreements between the two authors (JS and MH) were discussed, where after a third independent assessor (GK) was involved to reach consensus.

### Data extraction and statistical analysis

The following data were extracted from the studies: 1) authors and year of publication; 2) sample size; 3) participant information, including age, gender, stroke etiology, location of stroke, and severity of UL-motor deficit; 4) details of TMS-MEP testing, including timing after stroke, target muscle, coil location, and intensity of stimulation, and 5) outcome measures, including timing of assessment and cut-off values used. We harmonized MEP outcomes across studies by adopting the authors’ original binary classifications where available, and for studies reporting numerical MEP values, any detectable response on TMS parameters (e.g., amplitude or latency) was considered positive, while measurements with no observable response (“0”) were considered negative.

To evaluate TMS-MEP as a prognostic biomarker (35), each included study needed to provide at least four values: the number of true positives (TP), false positives (FP), true negatives (TN), and false negatives (FN), allowing for the reconstruction of a two-by-two table. Meta-analysis was primarily conducted using aggregated data at the group level. For the outcome measures, individual patient data were used to determine cut-off values when they were available, as different cut-off values may have been used across studies. Favorable outcomes were defined as the ability to perform UL movements against gravity, corresponding to MRC scores of ≥3, or equivalent thresholds on other outcome scales.

We used the online MetaDTA tool for the statistical analysis (36, 37). A Bivariate random-effects model was employed to calculate the pooled sensitivity, specificity, positive predictive value (PPV), negative predictive value (NPV), and diagnostic odds ratio (DOR), along with their 95% confidence intervals (CIs).

In this study, sensitivity refers to the probability that TMS-MEP correctly identifies all patients with UL-motor recovery who have a positive TMS-MEP at baseline. Specificity indicates the probability that TMS-MEP correctly identifies all patients who do not experience UL-motor recovery and have an absent TMS-MEP at baseline. PPV indicates how likely it is that patients with a positive TMS-MEP at baseline, truly had UL-motor recovery. Conversely, the NPV refers to the probability that TMS-MEP correctly identifies patients without TMS-MEP who do not have UL-motor recovery (38). The DOR calculates the ratio of the odds of a positive TMS-MEP in a patient with UL-motor recovery compared to the odds of a positive TMS-MEP in a patient without UL-motor recovery. Higher DOR values indicate better performance of the diagnostic test (39). Additionally, we used a hierarchical summary receiver operating characteristic (HSROC) model to evaluate the overall diagnostic test accuracy (DTA) from the included studies (37). In the HSROC model, the Summary ROC (SROC) curve represents the overall diagnostic performance across studies, accounting for between-study heterogeneity in sensitivity and specificity, summarized by the 95% prediction region.

IBM SPSS version 28 was used to test for differences between prespecified groups based on timing (baseline TMS MEPs ≤7 days versus >7 days) and on outcome construct (muscle strength, muscle synergy and UL-capacity), using the non-parametric Kruskal-Wallis test. Statistical significance was defined as a two-tailed *p* < 0.05.

## Results

### Search results

**Figure 1:**
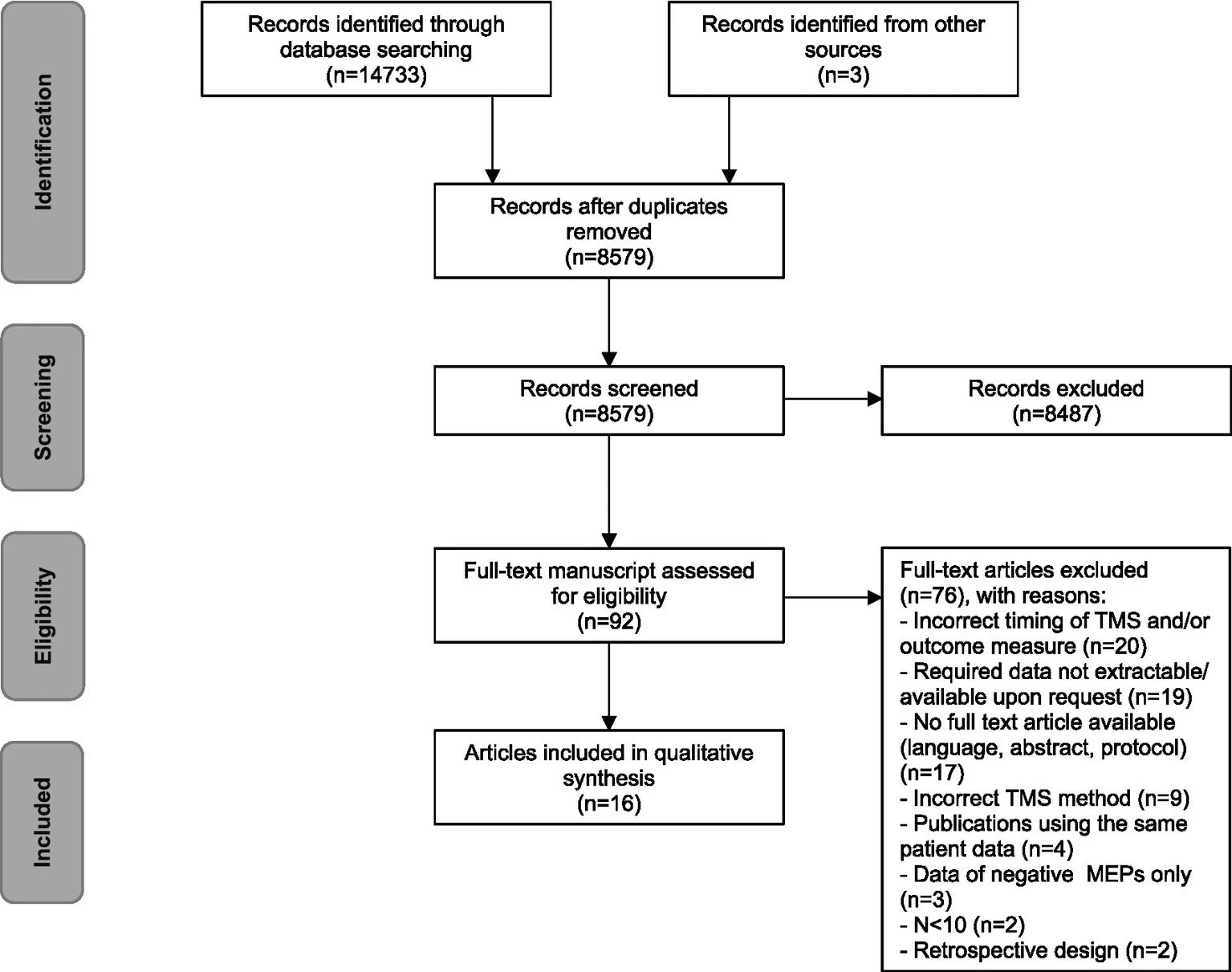
Flowchart outlining the search and selection process for the studies.

Figure 1 shows the flow chart of references included in this review. As shown, the literature search revealed a total of 14.733 references, which included 2.663 from PubMed, 4.559 from Embase, 1.040 from PsycInfo, 826 from CINAHL, 3.713 from Web of Science, 1.679 from Cochrane, and 253 from ICTRP. As illustrated, we removed 6.154 duplicate references, resulting in 8.579 potential references for review. After screening the abstracts, we selected 92 studies for further evaluation. However, we were unable to extract individual data from the manuscripts of 23 of these studies. We contacted the corresponding authors of these studies to request the specific data needed for our analysis, and four authors provided the requested information (7, 40–42). A total of 16 studies were ultimately included in the present meta-analysis.

### Description of the studies

Table 1 presents an overview of the included studies. In total, 556 participants of 16 unique studies were included. The sample sizes across the studies ranged from 10 to 71 participants. The majority suffered from ischemic stroke, while 78 participants (14%) had a hemorrhagic stroke. The mean age of the participants ranged from 51.5 to 71.6 years. Out of the clinical data available for 484 subjects, 291 (60%) participants achieved a favorable UL-motor function outcome, whereas 193 subjects were classified as having a low UL-motor score based on the outcome measures and cut-off scores used in each study.

**Table 1:** Characteristics of the included studies.

Authors	Initial sample size (N)	Stroke etiology ischemic/hemorrhagic	First-ever stroke	Right/left hemisphere (N)	Mean age (years)	Gender F/M (N)	Timepoint TMS-testing (d ± SD)	Target muscle	Outcome measures	Outcome timing (w)
Arac et al. (50)	27	19/8	Unknown	16/11	62.5 range 29–79	13/14	< 7	APB	MRC (‘hand muscle’)	13–26
Bakker et al. (41)	19	19/0	Yes	7/12	63.8 range 45–80	7/12	< 14	ADM	FMA-UE	26
Bembenek et al. (7)	71	68/3	Yes	33/38	65.3 range 26–83	26/45	< 14	ADM	MBC; MRC (“hand muscle”)	13
Cicinelli et al. (51)	18	18*	Yes	3/15	61.1 SD 12	6/12	53 ± 7	ADM	CNS-hms	13–15
Cruz Martinez et all. (52)	20	20/0	Unknown	14/5, 1 brainstem	56.9 SD 10.4	5/15	< 6	Thenar	CNS-hms	26
Delvaux et al. (53)	31	31/0	Yes	8/8	65.3 range 17–91	15/16	1	FDI	MRC (‘hand muscle’)	52
Freundlieb et al. (54)	12	12/0	Yes	5/5	68 range 37–88	3/9	< 5	FDI	FMA-UE, NHPT	26
Hendricks et al. (55)	29	29/0	Yes	Unknown	63.7 range 22–85	15/14	< 4	ADM	FMA-UE	52–208
Hendricks et al. (56)	43	43/0	Unknown	Unknown	66.9 range 19–84	22/21	< 10	ADM	FMA-hand, FAT	26
Hoonhorst et al. (42)	51	51/0	Yes	26/25	70 range 44–93	21/30	< 2	ADM	FMA-UE	26
Jang et al. (57)	53	53/0	Yes	28/25	54 range 41–79	22/31	17 ± 3	APB	MBC, MI (arm)	26
Kumar et al. (58)	29	29/0	Yes	12/17	51.5 SD 11.3	8/21	< 7	APB	ARAT	13
Pizzi et al. (59)	52	32/20	Yes	31/21	62 range 21–86	25/27	33	ADM	MRC (ADM), FAT	52
Schambra et al. (40)	45	0/45	Yes	29/16	60 range 22–69	14/31	11 ± 3.6	FDI	FMA-UE	26
Trompetto et al. (60)	21	16/5	Yes	14/7	71.6 range 39–90	9/12	< 5	Thenar	SSS-hms	26
van Kuijk et al. (61)	35	35/0	Yes	15/20	59.8 range 20–86	18/17	7	ADM	FMA-hand	26

ADM, adductor digiti minimi muscle; APB, abductor pollicis brevis muscle; ARAT, Action Research Arm Test; CNS, Canadian neurological scale hand-motor-score; FAT, Frenchay Arm Test; FDI, flexor digitorum interossei muscle; FMA-hand, Fugl meyer assessment hand scores; FMA-UE, Fugl meyer assessment of the Upper Extremity; MBC, Modified Brunnstrom Classification; MI, Motricity index arm sub-score; MRC, Medical research council; NHPT, nine-hole peg test; SSS, Scandinavian stroke scale hand-motor-score; TMS-MEP, transcranial magnetic stimulation-induced motor evoked potentials.

*Unknown.

### TMS-MEP assessment overview

Of the 484 participants that underwent TMS-MEP assessment within eight weeks following stroke, 243 participants had a positive TMS-MEP, while 241 displayed absent TMS-MEP responses. Nine studies conducted the TMS-MEP assessment within or at seventh days post-stroke. The most often used muscle for TMS-MEP evaluation was the Abductor Digiti Minimi (ADM) (*n* = 8), followed by the First Dorsal Interossei (FDI) (*n* = 3) and the Abductor Pollicis Brevis (APB) muscle (*n* = 3).

### Outcome measures in the included studies

A total of nine different outcome measures for UL-motor function were utilized across the 16 studies reviewed, as presented in Table 2. The most frequently used outcome measures were the FMA-upper extremity (UE) or FMA-hand (*n* = 7) and the MRC of the hand muscles (*n* = 4).

**Table 2:** Summary of the types of outcome measures used in the included studies to define upper limb motor outcomes.

Construct	Outcome scale	Abbreviation	Cut-off value
Muscle strength	Canadian neurological scale, hand motor score	CNS-hms	≥0.5
	Medical research council, muscle strength	MRC	≥3
	Motricity index, upper limb score	MI	≥62*
	Scandinavian stroke scale, hand motor score	SSS-hms	≥4
Muscle synergy	Fugl-meyer motor assessment, upper extremity	FMA-UE	>23
	Fugl-meyer motor assessment, hand	FMA-hand	≥4*
UL capacity	Action research arm test	ARAT	≥10*
	Frenchay arm test	FAT	≥2
	Modified brunnstrom classification	MBC	≥3*
	Nine hole peg test	NHPT	>0

This overview outlines the types of motor outcome measures used, along with their cut-off scores that define favorable outcomes. Specifically, the ability to perform movements against gravity is assessed with a Motor Recovery Classification (MRC) score of ≥3, or equivalent scores on other measures. The outcomes are categorized into three distinct groups.

^*^Cut-off scores are derived from the original study due to the unavailability of individual patient data.

### The methodological quality and reporting of the included studies

**Figure 2:**
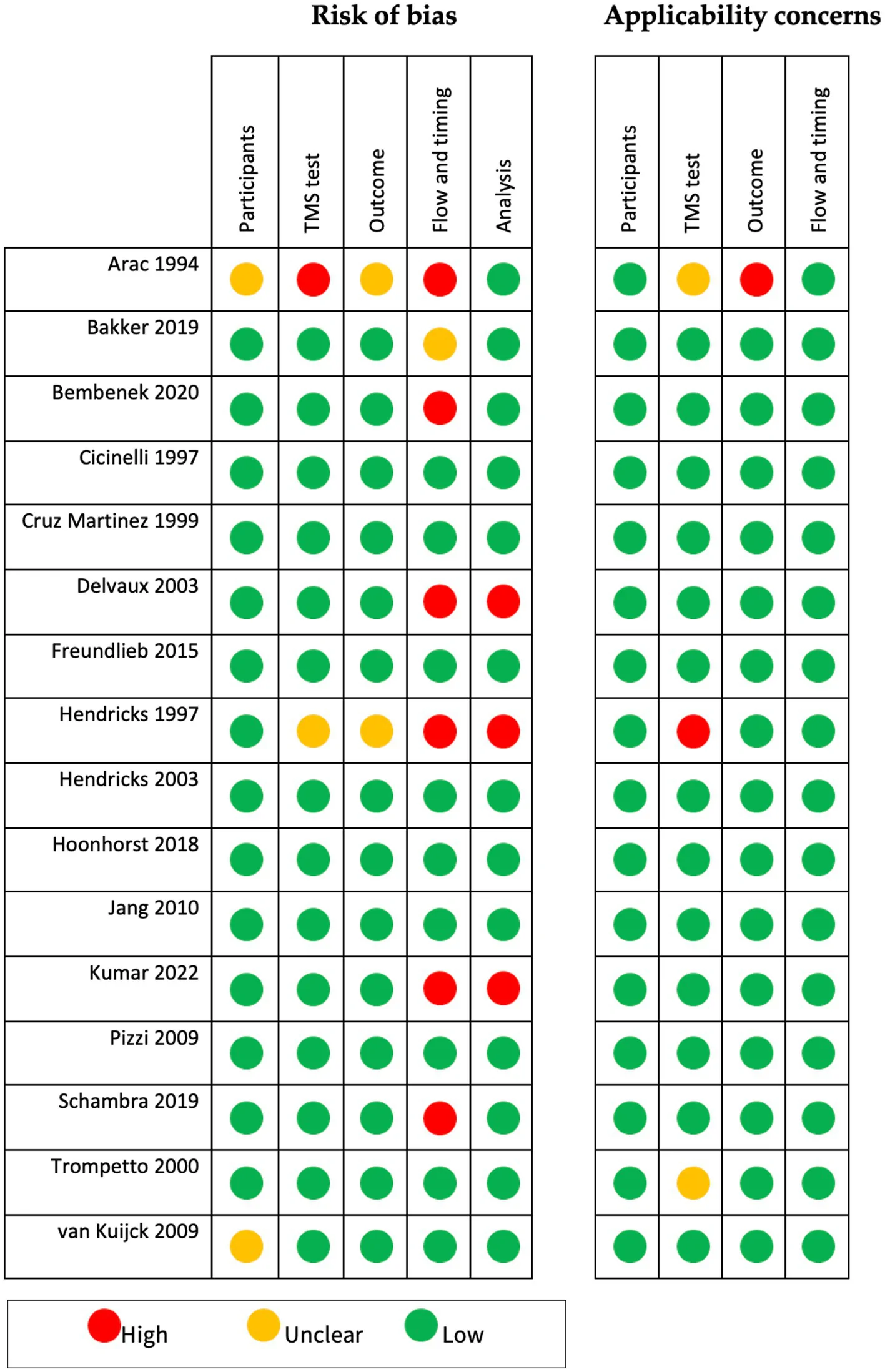
Graphical summary of risk of bias and applicability concerns from the included TMS-MEP studies, as assessed by the QUAPAS tool.

As illustrated in Figure 2, most studies exhibited a low RoB and posed minimal concerns regarding their applicability. However, six studies were identified as having a high RoB in the QUAPAS domain 4. This was primarily due to missing or incomplete data, as well as significant rates of loss to follow up. According to the revised Chipchase checklist, although 11 studies reported at least 75% of selected items, no studies reported all relevant information. For instance, as outlined in Supplementary Material, Section S3, nine studies did not specify how the hotspot was determined, eleven studies omitted the methods used to establish the MEP threshold, and fourteen studies did not report on possible prescribed central nervous system (CNS) active medications for the subjects.

### Quantitative analyses of the included studies

**Figure 3:**
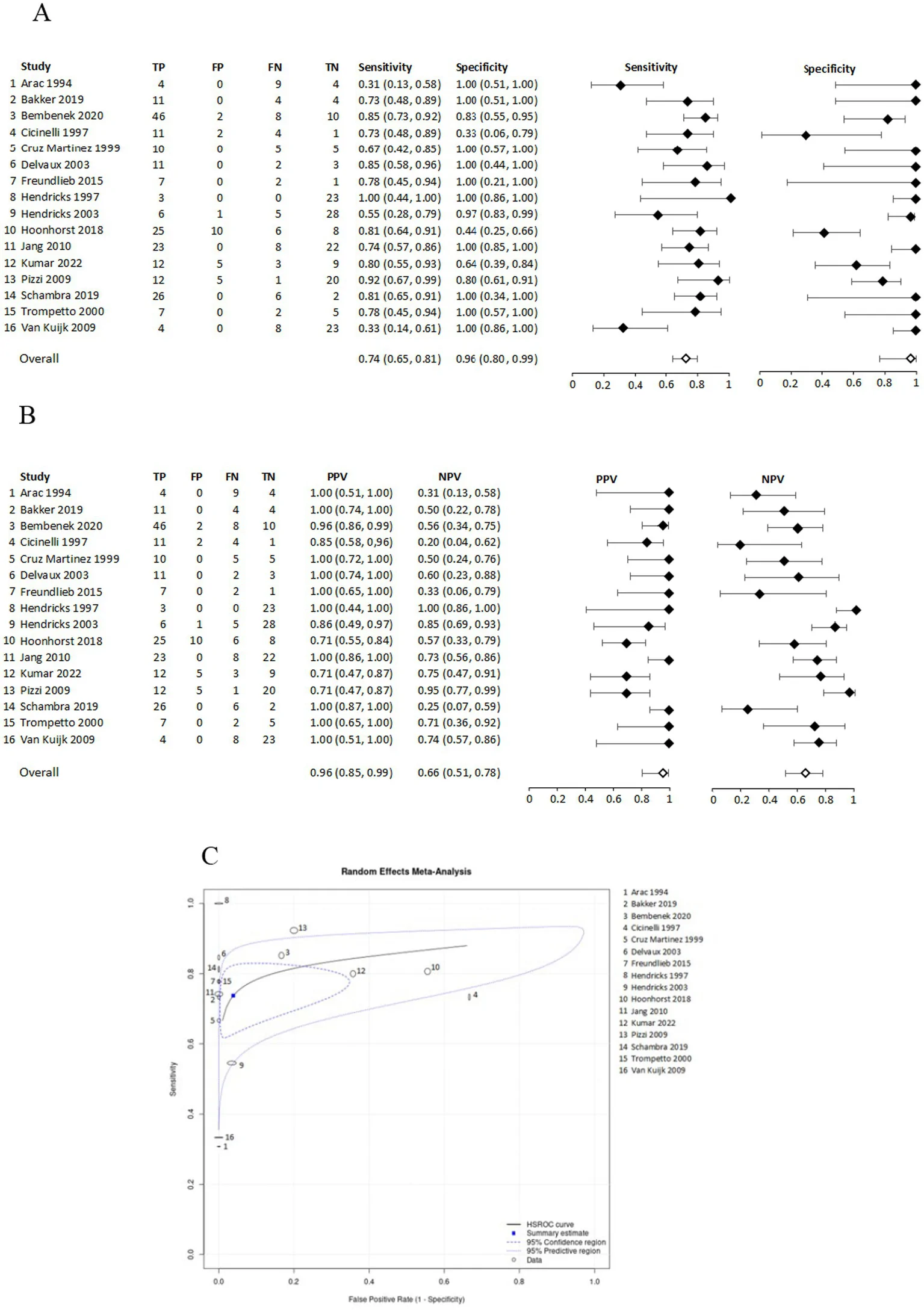
Forest Plots and the hierarchical summary receiver operating characteristic curve (HSROC) plot for transcranial magnetic stimulation motor-evoked potentials predicting upper limb motor function outcomes in stroke patients. The forest plots illustrate the extracted data from each study, presenting estimates of sensitivity and specificity **(A)**, as well as the positive predictive value (PPV) and negative predictive value (NPV) **(B)**, accompanied with 95% confidence intervals. The pooled sensitivity was 0.74 (95% CI: 0.65-0.81), while the pooled specificity was 0.96 (95% CI: 0.80 0.99). The calculated diagnostic odds ratio (DOR) was 70.1 (95% CI: 12.3-402.5). The pooled PPV was 0.96 (95% CI: 0.85-0.99), and the pooled NPV was 0.66 (95% CI: 0.51-0.78). The HSROC plot is shown in **(C)**. The solid black line represents the summary receiver operating characteristic (SROC) curve, while the blue square indicates the summary estimate. The fine blue dotted line defines the 95% prediction region. Each individual study estimate is represented as a black open circle, with the size of the circles corresponding to each study's weight in the meta-analysis (37). The height and width of the circles vary in proportion to these study weights. FN (false negatives), FP (false positives), TN (true negatives), TP (true positives).

As illustrated in Figure 3, the pooled sensitivity of TMS-MEP was 0.74 (95% CI: 0.65–0.81), and the specificity was 0.96 (95% CI: 0.80–0.99). This resulted in a DOR of 70.1 (95% CI: 12.3–402.5). The pooled PPV was 0.96 (95% CI: 0.85–0.99), while the NPV was 0.66 (95% CI: 0.51–0.78). On the HSROC curve, studies with more extreme values for sensitivity or specificity, located on the left-hand side of Figure 3, were smaller in size, resulting in a lower weight in the HSROC curve plot. The 16 studies had a median prevalence of 0.70 (IQR 0.39–0.82).

### Timing of TMS-MEP assessment and type of upper limb motor outcomes

**Figure 4:**
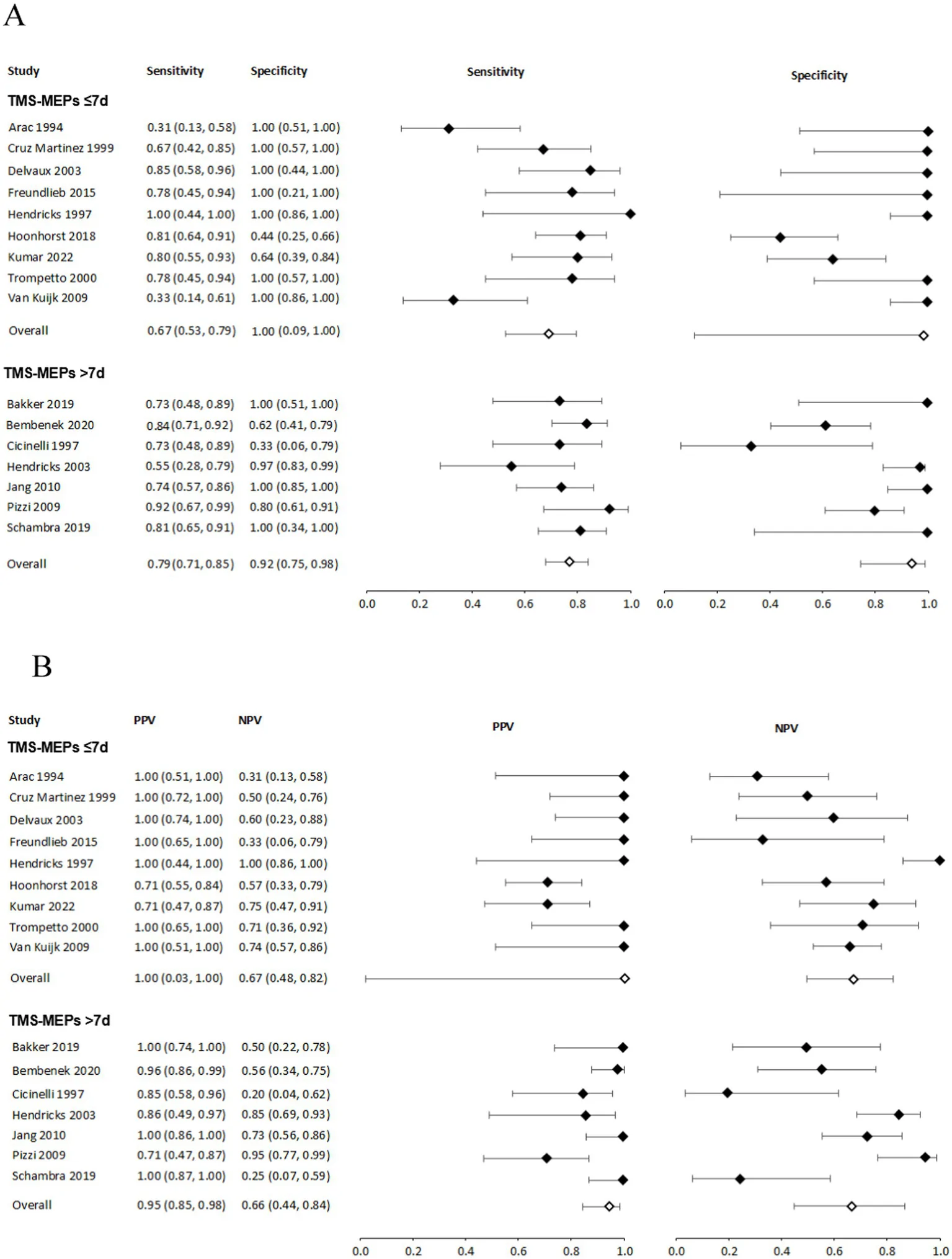
Illustration of forest plots from the subgroup analysis based on the timing of baseline TMS measurements. The forest plots display the extracted data from each study and focus on the timing of the baseline TMS measurement. They include estimates for sensitivity, specificity **(A)**, as well as for positive predictive value (PPV) and negative predictive value (NPV) **(B)**. For studies that conducted baseline TMS measurements on or before the seventh day, the pooled sensitivity was 0.67 (95% CI: 0.53–0.79), and the pooled specificity was 1.00 (95% CI: 0.09–1.00). The diagnostic odds ratio (DOR) for these studies was 573 (95% CI: 0.24–1,361,960). The pooled PPV was 1.00 (95% CI: 0.03–1.00), while the NPV was 0.67 (95% CI: 0.48–0.82). In contrast, for studies that performed a baseline TMS measurement after the seventh day, the pooled sensitivity increased to 0.79 (95% CI: 0.71–0.85), and the pooled specificity was 0.92 (95% CI: 0.75–0.98). The DOR for these studies was 40.4 (95% CI: 11.8–138.6). The pooled PPV was 0.95 (95% CI: 0.85–0.98), and the NPV was 0.66 (95% CI: 0.44–0.84).

**Figure 5:**
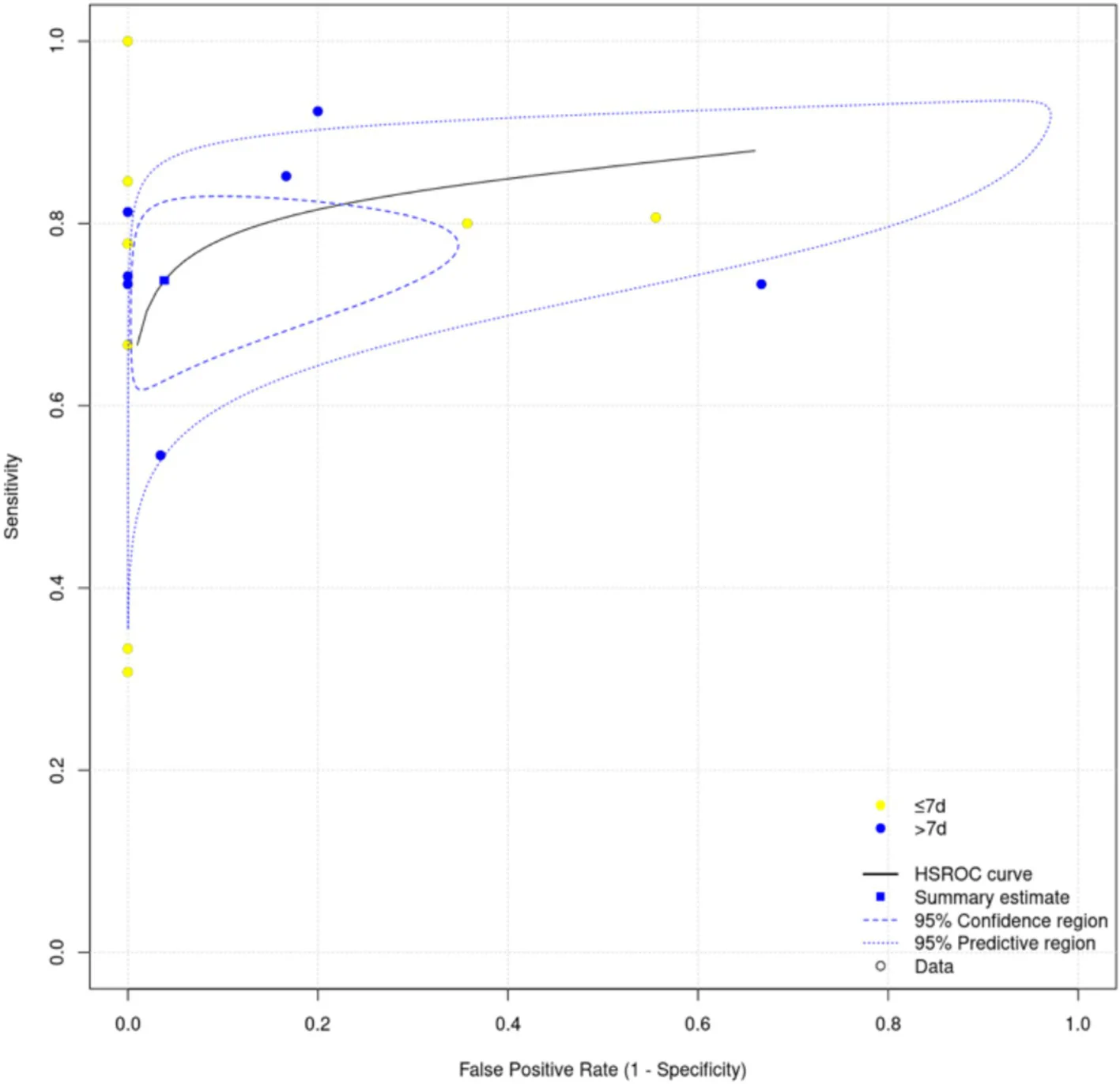
Display of a hierarchical summary receiver operating characteristic (HSROC) curve that illustrates the prognostic accuracy of transcranial magnetic stimulation (TMS) motor-evoked potentials (MEPs) in predicting upper limb motor outcomes. The data is categorized according to the timing of the baseline TMS measurement. The solid black line represents the summary receiver operating characteristic (SROC) curve. Yellow dots indicate individual studies where TMS measurements were taken before or on the seventh day post-stroke. In contrast, blue dots represent studies with TMS measurements conducted after the seventh day. The filled blue square marks the summary estimate, while the fine blue dotted curve illustrates the 95% prediction region.

As illustrated in Figures 4, 5, for studies where the baseline TMS-MEP assessment was conducted before or on the seventh day, the pooled sensitivity was 0.67 (95% CI: 0.53–0.79) and the specificity was 1.00 (95% CI: 0.09–1.00), resulting in a DOR of 573 (95% CI: 0.24–1,361,960). The pooled PPV was 1.00 (95% CI: 0.03–1.00), while the pooled NPV was 0.67 (95% CI: 0.48–0.82). In contrast, for studies where the baseline TMS-MEP assessment was conducted after the seventh day, the pooled sensitivity increased to 0.79 (95% CI: 0.71–0.85) while the specificity decreased to 0.92 (95% CI: 0.75–0.98), resulting in a DOR of 40.4 (11.8–138.6). The pooled PPV in this case was 0.95 (95% CI: 0.85–0.98) and the NPV was 0.66 (95% CI: 0.44–0.84).

**Figure 6:**
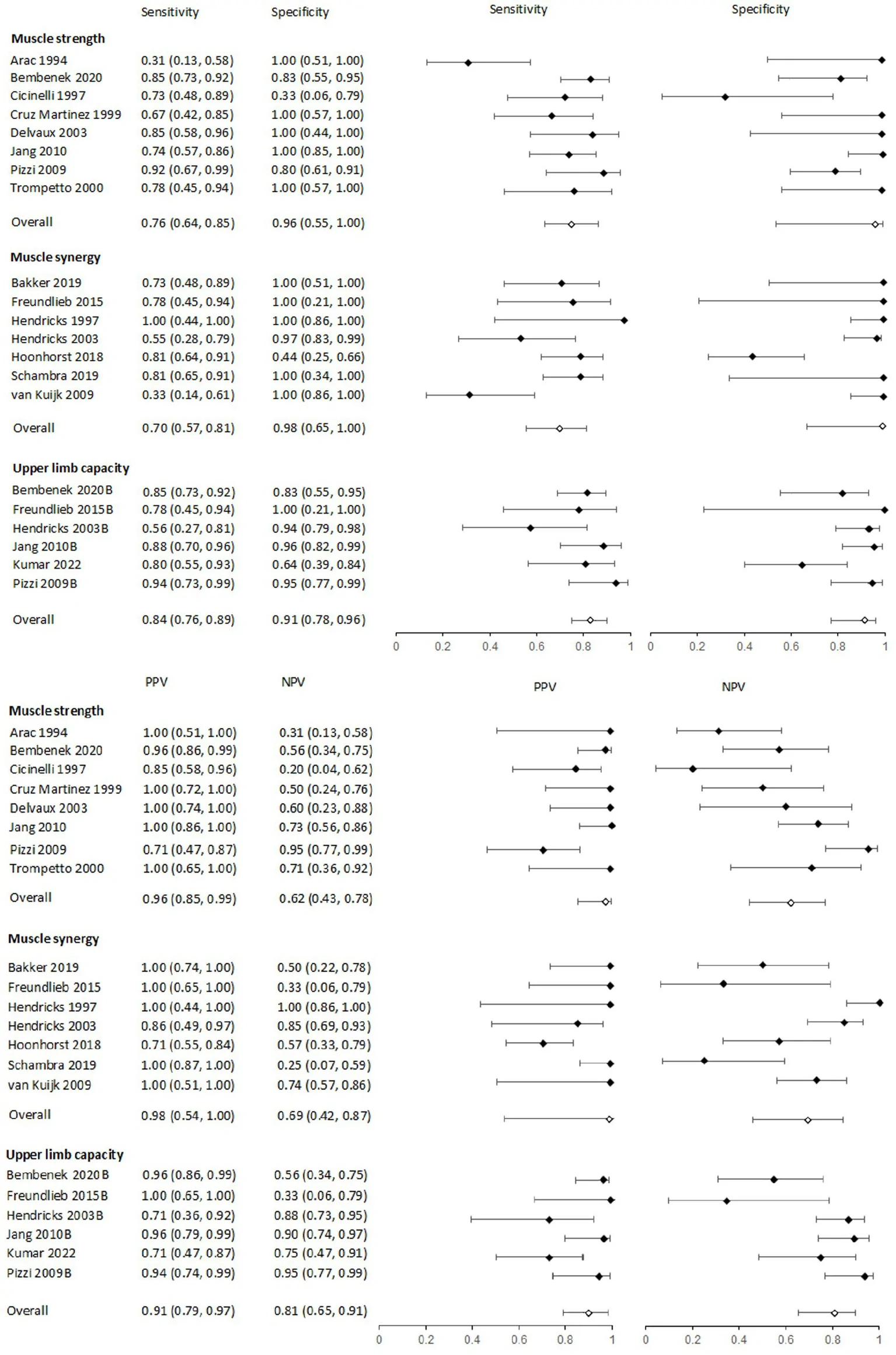
Forest plots from a subgroup analysis categorized by different types of upper limb (UL) motor function outcomes: muscle strength, muscle synergy, and UL-capacity. In studies focused on muscle strength, the pooled sensitivity was 0.76 (95% CI: 0.64–0.85), and the pooled specificity was 0.96 (95% CI: 0.55–1.00). The diagnostic odds ratio (DOR) was 79.6 (95% CI: 4.63–1371.2). Additionally, the pooled positive predictive value (PPV) was 0.96 (95% CI: 0.85–0.99), while the negative predictive value (NPV) was 0.62 (95% CI: 0.43–0.78). For the studies assessing muscle synergy, the pooled sensitivity was 0.70 (95% CI: 0.57–0.81), and the pooled specificity was 0.98 (95% CI: 0.65–1.00). The DOR for muscle synergy was 101.5 (95% CI: 4.88–2113.6). The pooled PPV was 0.98 (95% CI: 0.54–1.00), and the NPV was 0.69 (95% CI: 0.42–0.87). In studies evaluating UL-capacity, the pooled sensitivity was 0.84 (95% CI: 0.76–0.89), while the pooled specificity was 0.91 (95% CI: 0.78–0.96). The DOR was 49.1 (95% CI: 15.8–153.1). Additionally, the pooled PPV was 0.91 (95% CI: 0.79–0.97), and the NPV was 0.81 (95% CI: 0.65–0.91).

Figure 6 illustrates the results for muscle strength assessment, revealing a sensitivity of 0.76 (95%CI: 0.64–0.85) and a specificity of 0.96 (95% CI: 0.55–1.00). The PPV was 0.96 (95% CI: 0.85–0.99), while the NPV was 0.62 (95% CI: 0.43–0.78). Additionally, the DOR was 79.6 (95% CI: 4.63–1371.2). In studies evaluating muscle synergy, the pooled sensitivity was found to be 0.70 (95% CI: 0.57–0.81) and the specificity was 0.98 (95%CI: 0.65–1.00), resulting in a DOR of 101.5 (95% CI: 4.88–2113.6). The pooled PPV was 0.98 (95%CI: 0.54–1.00), with a NPV 0.69 (95% CI: 0.42–0.87). For studies measuring UL-capacity, the pooled sensitivity was 0.84 (95% CI: 0.76–0.89), and the specificity was 0.91 (95% CI: 0.78–0.96), resulting in a DOR of 49.1 (95% CI: 15.8–153.1). The pooled PPV for these studies was 0.91 (95% CI: 0.79–0.97), while the NPV was 0.81 (95% CI: 0.65–0.91).

Finally, non-parametric analysis using the Kruskal-Wallis test showed no significant differences between outcome types (i.e., muscle strength, muscle synergy and UL-capacity) in terms of sensitivity (*p* = 0.53), specificity (*p* = 0.32), PPV (*p* = 0.24) and NPV (*p* = 0.41). Similarly, no significant differences were found between subjects who were assessed within or beyond the first 7 days with respect to overall sensitivity (*p* = 0.92), specificity (*p* = 0.30), PPV (*p* = 0.41) and NPV (*p* = 0.68).

## Discussion

This research reviewed technical recommendations and methodological quality in studies using TMS-MEP to predict upper limb muscle strength, synergies, and capacity after stroke. Despite some omissions, the methodology was adequate; however, the small sample sizes of included studies led to imprecise estimates and wide confidence intervals. Regarding the technical aspects of those 16 studies, information was lacking on key areas such as hotspot determination (*n* = 9), MEP thresholds (*n* = 11), and CNS medication use among subjects (*n* = 14). While the number of technical execution items reported showed an upward trend over time of publication, it remains unclear how the execution was technically performed.

Meta-analysis of data from 484 patients across the included studies showed that early diagnostic TMS-MEPs have a high pooled PPV [0.96 (0.85–0.99)] and specificity [0.96 (0.80–0.99)]. The high pooled PPV emphasizes that the presence of TMS-MEPs at baseline is a valid predictor for identifying subjects with a favorable upper limb recovery, which is consistent with findings from studies not included in this meta-analysis, such as the PREP1 (43) and PREP2 (16, 44) and our previous published TMS-ADM MEP models (42). Although the surplus value of TMS-MEP compared to clinical testing such as using SAFE scores is still under discussion when applied at the same time post-stroke (42), it should be noted that, unlike clinical modelling, TMS-MEP can provide useful information when clinical testing is uncertain, such as in cases of apraxia, motor neglect, or when the subject has communicative or cognitive problems.

Of greater relevance is the finding that TMS-MEPs are less accurate in predicting motor outcomes when the initial MEP is negative, as shown by the consistently low NPVs. These findings align with previous clinical studies (e.g., Nijland et al. (6)) and TMS-MEP studies [e.g., Hoonhorst et al. (42)], although many prior studies included a relatively small proportion of severely impaired patients with stroke. This underrepresentation in most studies introduces greater imprecision in current TMS-MEP research and raises concerns regarding the generalizability of findings to the most severely affected individuals. In our study, we have confirmed low NPV values in a large cohort of initial MEP-negative patients (50%) and a substantial proportion of poor recoverers (40%). These results suggests that, even with large sample sizes, the predictive accuracy of TMS-MEPs remains relatively low. Importantly, this points to a fundamental challenge in predicting recovery in those patients with low baseline motor scores and negative MEPs, who may still demonstrate potential for UL recovery in the first 3 months post-stroke. The causes underlying the excessively pessimistic predictions associated with TMS-MEPs remain unclear and warrant further investigation. It is possible that the low NPV is partly attributable to technical limitations inherent to TMS methodology, such as incorrect coil placement, suboptimal stimulation intensity, or issues with recording of the evoked muscle response, all of which may result in absent MEPs (45–47). Beyond these technical factors, it is also plausible that other factors such as diaschisis or shock causing functional suppression of anatomically-associated neural networks (48), play a role in the relatively large number of false negatives early after stroke. In such cases, the absence of early MEPs may reflect reversible factors, such as vasogenic edema, neural inflammation, or shock, rather than irreversible damage to the CST itself (49).

In contrast to our hypothesis, the present meta-analysis found no significant effect of baseline TMS-MEP measurement timing on prognostic accuracy, with similar results for assessments before or on day 7 post-stroke and those after day 7. Although later measurements were expected to show higher sensitivity and NPV, differences were not statistically significant. This lack of significance between pooled studies within and beyond the first seven days may be caused by the large between-study variance as well as the limited number of small-sampled studies in our meta-analysis.

Finally, we hypothesized that post-stroke prognostic values may differ due to varying outcome measures of motor function. While the UL-capacity group showed slightly higher sensitivity, specificity, NPV, and PPV than measures reflecting muscle synergies (such as FMA-UE) or muscle strength (such as MRC, MI), the differences were not statistically significant, suggesting that these measures can be used interchangeably.

### Study limitations

This review is subject to limitations related to missing data, which may have affected our results. Of the initial sample of 556 patients, 72 subjects had missing data for analysis. This attrition could have introduced bias, particularly if missingness was associated with poorer outcomes, and, even if random, the reduced sample size may have limited statistical power. Additionally, 19 studies meeting our inclusion criteria were excluded due to the unavailability of relevant data, despite attempts to obtain these from the corresponding authors. As these excluded studies involved patient populations and methodologies comparable to those included, the risk of systematic bias appears limited, yet their absence may have further reduced the overall sample size and statistical power. Adherence to the FAIR (Findable, Accessible, Interoperable, and Reusable) principles for scientific data management (49) could have mitigated these limitations by enhancing the accessibility, comparability, and reusability of data, ultimately improving understanding of the prognostic value of early post-stroke TMS-MEP.

Careful interpretation of the pooled prognostic accuracy is required due to substantial heterogeneity across studies, arising from differences in patient populations, stroke severity, TMS stimulation parameters, outcome measures, and follow-up durations. By selecting studies with consistent timing of baseline measurements and specific motor function outcomes, we reduced some key sources of variability, yet considerable heterogeneity persists. The bivariate HSROC model accounts for variability in sensitivity and specificity, but the wide 95% prediction region (Figures 3, 5) underscores that substantial heterogeneity remains and results may still vary between studies.

## Conclusion

Early positive TMS-MEP results are associated with a high positive predictive value for motor recovery potential. However, in patients without detectable TMS-MEPs initially, predictive accuracy is limited, highlighting the challenge of early prognostication in this subgroup. Prognostic accuracy may be improved by combining TMS-MEPs with other biomarkers in a multimodal approach, a promising direction for future research. Methodological differences and missing data highlight the importance of standardized TMS protocols and thorough data collection, ideally following the FAIR principles (49). Overall, clinicians should recognize that current prognostic biomarkers may not fully capture recovery potential early post-stroke, particularly in severely affected patients who can still benefit from intensive rehabilitation.

## Data Availability

The original contributions presented in the study are included in the article/Supplementary material, further inquiries can be directed to the corresponding author.
