# Correction: Prognostic accuracy of transcranial magnetic stimulation-induced motor evoked potentials on recovery of upper limb: a systematic review

**DOI:** 10.3389/fneur.2026.1967234

**Published:** 2026-09-02

**Authors:** Johanna C. M. Schilder, Maurits H. J. Hoonhorst, Ralph de Vries, Gert Kwakkel

**Affiliations:** 1 Department of Rehabilitation Medicine, Amsterdam UMC Location University of Amsterdam, Amsterdam, Netherlands; 2 Department of Neurorehabilitation, Rehabilitation Centre Vogellanden, Zwolle, Netherlands; 3 Medical Library, Amsterdam UMC Location Vrije Universiteit Amsterdam, Amsterdam, Netherlands; 4 Department of Neurorehabilitation, Amsterdam Rehabilitation Research Centre Reade, Amsterdam, Netherlands; 5 Department of Physical Therapy and Human Movement Sciences, Northwestern University, Chicago, IL, United States; 6 Department of Rehabilitation Medicine, Amsterdam UMC Location Vrije Universiteit Amsterdam, Amsterdam Neurosciences and Amsterdam Movement Sciences, Amsterdam, Netherlands

**Keywords:** corticospinal tract, motor evoked potential, motor recovery, prognostic accuracy, stroke, transcranial magnetic stimulation, upper extremity

In the published article, reference no. 16 “Stinear et al. (2017)” was erroneously added in the third paragraph of the Discussion section, and has therefore been removed.

Below is the full reference: “Stinear CM, Byblow WD, Ackerley SJ, Smith MC, Borges VM, Barber PA. PREP2: a biomarker-based algorithm for predicting upper limb function after stroke. *Ann Clin Transl Neurol*. (2017) 4:811–20”.

The original version of this article has been updated.

