# Supplementary material for: Prognostic accuracy of transcranial magnetic stimulation-induced motor evoked potentials on recovery of upper limb: a systematic review
Source: Front Neurol. 2026 May 7;17:1788829. doi: 10.3389/fneur.2026.1788829 (PMC13192014; doi:10.3389/fneur.2026.1788829)
Supplement: Supplementary file 1 [file Supplementary_File_1.docx]

## Supplementary material Section 1.

### Full search strategies for all databases and registers.

PubMed Session results (25 May 2023):

| **#** | **Query** | **Results** |
| --- | --- | --- |
| **#3** | #1 AND #2 | **2,663** |
| **#2** | "Stroke"[Mesh] OR "Stroke Rehabilitation"[Mesh] OR stroke[tiab] OR strokes[tiab] OR "cerebrovascular accident*"[tiab] OR CVA [tiab] OR CVAs[tiab] | **361,754** |
| **#1** | "Transcranial Magnetic Stimulation"[Mesh] OR "Evoked Potentials, Motor"[Mesh] OR "Transcranial Magnetic Stimulation*"[tiab] OR "Motor Evoked Potential*"[tiab] OR TMS [tiab] OR MEP [tiab] | **39,792** |

Embase.com Session results (25 May 2023):

| **#** | **Query** | **Results** |
| --- | --- | --- |
| **#3** | #1 AND #2 | **4,559** |
| **#2** | 'cerebrovascular accident'/exp OR stroke:ab,ti,kw OR strokes:ab,ti,kw OR 'cerebrovascular accident*':ab,ti,kw OR CVA:ab,ti,kw OR CVAs:ab,ti,kw | **621,643** |
| **#1** | 'transcranial magnetic stimulation'/exp OR 'motor evoked potential'/exp OR 'transcranial magnetic stimulation*':ab,ti,kw OR 'motor evoked potential*':ab,ti,kw OR TMS:ab,ti,kw OR MEP:ab,ti,kw | **58,200** |

APA PsycInfo (Ebsco) Session results (25 May 2023):

| **#** | **Query** | **Results** |
| --- | --- | --- |
| **#3** | #1 AND #2 | **1,040** |
| **#2** | DE "Cerebrovascular Accidents" OR TI (stroke OR strokes OR "cerebrovascular accident*" OR CVA OR CVAs) OR AB (stroke OR strokes OR "cerebrovascular accident*" OR CVA OR CVAs) OR KW (stroke OR strokes OR "cerebrovascular accident*" OR CVA OR CVAs) | **41,976** |
| **#1** | DE "Transcranial Magnetic Stimulation" OR DE "Somatosensory Evoked Potentials" OR TI ("transcranial magnetic stimulation*" OR "motor evoked potential*" OR TMS OR MEP) OR AB ("transcranial magnetic stimulation*" OR "motor evoked potential*" OR TMS OR MEP) OR KW ("transcranial magnetic stimulation*" OR "motor evoked potential*" OR TMS OR MEP) | **16,146** |

CINAHL (Ebsco) Session results (25 May 2023):

| **#** | **Query** | **Results** |
| --- | --- | --- |
| **#3** | #1 AND #2 | **826** |
| **#2** | (MH "Stroke+") OR TI (stroke OR strokes OR "cerebrovascular accident*" OR CVA OR CVAs) OR AB (stroke OR strokes OR "cerebrovascular accident*" OR CVA OR CVAs) OR SU (stroke OR strokes OR "cerebrovascular accident*" OR CVA OR CVAs) | **142,545** |
| **#1** | (MH "Transcranial Magnetic Stimulation") OR (MH "Evoked Potentials, Motor") OR TI ("transcranial magnetic stimulation*" OR "motor evoked potential*" OR TMS OR MEP) OR AB ("transcranial magnetic stimulation*" OR "motor evoked potential*" OR TMS OR MEP) OR SU ("transcranial magnetic stimulation*" OR "motor evoked potential*" OR TMS OR MEP) | **7,516** |

Web of Science (Core Collection) Session results (25 May 2023):

| **#** | **Query** | **Results** |
| --- | --- | --- |
| **#3** | #1 AND #2 | **3,713** |
| **#2** | TS=("stroke" OR "strokes" OR "cerebrovascular accident*" OR "CVA" OR "CVAs") | **434,508** |
| **#1** | TS=("transcranial magnetic stimulation*" OR "motor evoked potential*" OR "TMS" OR "MEP") | **50,610** |

Cochrane Library (Wiley) Session results (25 May 2023):

| **#** | **Query** | **Results** |
| --- | --- | --- |
| **#3** | #1 AND #2 | **1,679** |
| **#2** | (stroke or strokes or (cerebrovascular NEXT accident*) or CVA or CVAs):ab,ti,kw | **71,182** |
| **#1** | ((transcranial NEXT magnetic NEXT stimulation*) or (motor NEXT evoked NEXT potential*) or TMS or MEP):ab,ti,kw | **8,852** |

WHO - ICTRP Session results (25 May 2023):

| **#** | **Query** | **Results** |
| --- | --- | --- |
| **#3** | #1 AND #2 | **253** |
| **#2** | (stroke OR strokes OR cerebrovascular accident* OR CVA OR CVAs) | **14,509** |
| **#1** | (Transcranial Magnetic Stimulation* OR Motor Evoked Potential* OR TMS OR MEP) | **2,529** |

## Supplementary material Section 2.

### Quality Assessment of Prognostic Accuracy Studies (QUAPAS) tool.

In our QUAPAS assessment, as presented in Table 1, we assessed five key domains to evaluate the risk of bias (RoB) and concerns regarding the applicability of our review questions, as outlined in the manuscript. Each domain contains specific signaling questions that guide the assessment of whether the RoB and applicability concerns are graded as low, high, or unclear. Every signaling question is accompanied by a rationale directly linked to our research questions. The signaling questions are designed so that a “Yes” response indicates a low RoB. If relevant information is not reported, the response is marked as "Unclear". Any signaling question rated as “No” indicates potential bias.

Concerns regarding applicability were similarly graded as low, high, or unclear, with low indicating minimal concern and high indicating substantial concern, reflecting the extent to which the participants, the TMS-MEP assessments, and clinical contexts of the included studies are relevant to and aligned with the review questions addressed in our analysis (1).

**Table 1** Additional signaling questions and rationales according to QUAPAS, supportive for the risk of bias judgement and applicability of the included studies.

|  | **Domain** | **Signaling questions** | **Rationales** |
| --- | --- | --- | --- |
| **Risk of Bias** | 1.Participants | Was a consecutive or random sample of participants enrolled? | Answer **“Yes”** if the study explicitly reports consecutive inclusion or random sampling. Answer **“No”** if participants were selected based on convenience, self-selection, or outcome status. Answer **“Unclear”** if the sampling strategy is not reported. |
|  |  | Was a case-control design avoided? | Answer **“Yes”** if participants were enrolled as a cohort of stroke patients prior to outcome ascertainment. Answer “No” if the text indicates a case-control design, includes multiple groups, or involves only healthy controls. Answer **“Unclear”** if the design is insufficiently described. |
|  |  | Did the study avoid inappropriate selection criteria? | Answer **“Yes”** if eligibility criteria are clearly stated and appropriate for the review question. Answer **“No”** if criteria are inappropriate or clearly introduce selection bias. Answer **“Unclear”** if eligibility criteria are incompletely reported. |
|  | 2. Index Test | Was the method used to perform the TMS-MEP assessment valid and reliable? | Answer **“Yes”** if TMS-MEP procedures are described in sufficient detail and are consistent with accepted methodological standards or guidelines (2,3). Answer **“No”** if methods are clearly inappropriate or invalid. Answer **“Unclear”** if reporting is insufficient to judge validity and TMS-MEP procedures. |
|  |  | Was the method for performing the TMS-MEP assessment the same for all participants? | Answer **“Yes”** if the same TMS-MEP protocol was applied to all participants. Answer **“No”** if different protocols were used without justification. Answer **“Unclear”** if protocol consistency cannot be determined. |
|  |  | Were the TMS-MEP results interpreted without knowledge of the outcome? | Answer **“Yes”** if TMS-MEP interpretation was performed blinded to clinical outcome data or occurred before outcome measurement. Answer **“No”** if interpretation of TMS-MEP results was performed with knowledge of the clinical outcome. Answer **“Unclear”** if blinding was not reported. |
|  |  | If a threshold was used, was it pre-specified? | Answer **“Yes”** if the prognostic cutoff (e.g., presence/absence of MEP, amplitude threshold) was defined a priori. Answer **“No”** if thresholds were data-driven or post hoc. Answer **“Unclear”** if threshold specification is not reported. |
|  | 3. Outcomes | Was the method used to measure the outcome valid and reliable? | Answer **“Yes”** if validated outcome measures for upper limb motor recovery were used. Answer **“No”** if outcomes are clearly inappropriate or prone to misclassification. Answer **“Unclear”** if outcome validity is not described. |
|  |  | Was the method for measuring the outcome the same for all participants? | Answer **“Yes”** if identical outcome measures and procedures were applied. Answer **“No”** if different methods were used. Answer **“Unclear”** if insufficient information is provided. |
|  |  | Was the outcome measured without knowledge of the index test results? | Answer **“Yes”** if outcome assessors were blinded to TMS-MEP test results. Answer **“No”** if blinding was explicitly not used. Answer **“Unclear”** if blinding is not reported. |
|  | 4. Flow and Timing | Did all participants receive the TMS-MEP assessment? | Answer **“Yes”** if all enrolled participants underwent TMS-MEP assessment. Answer **“No”** if some participants did not receive the assessment and data is missing. Answer **“Unclear”** if this cannot be determined. |
|  |  | Was treatment avoided after the TMS-MEP assessment was performed? | This signaling question in QUAPAS aims to address the potential for treatment selection bias. It recognizes that, in clinical practice, all patients usually undergo some form of rehabilitation, which makes it difficult to compare different treatment methods. A response of 'Yes' indicates that no participant received neurorehabilitation treatment between the TMS-MEP assessment and the outcome measurements. Answer 'No' if there is clear documentation showing that participants received neurorehabilitation during the study. If this information is ambiguous, answer 'Unclear'. |
|  |  | Was the time interval sufficient to capture the outcome on upper limb motor recovery? | Answer **“Yes”** if follow-up duration was sufficient to capture meaningful upper limb motor recovery (4,5). Answer **“No”** if follow-up was clearly inappropriate. Answer **“Unclear”** if timing is insufficiently reported. |
|  |  | Was information on the outcome available for all participants? | Answer **“Yes”** if loss to follow-up was minimal or appropriately addressed. Answer **“No”** if substantial missing outcome data were present without explanation. Answer **“Unclear”** if follow-up completeness cannot be determined |
|  | 5. Analysis | Were all enrolled participants included in the analysis? | Answer **“Yes”** if all participants were analyzed or exclusions were justified. Answer **“No”** if unexplained exclusions occurred. Answer **“Unclear”** if analysis of the population is unclear |
|  |  | If data were missing, were appropriate methods used? | Answer “Yes” if appropriate methods were used including any explanations regarding the reasons for and consequences of the identified missing data. Answer **“No”** if missing data were ignored. Answer **“Unclear”** if missing data were not reported. |
|  |  | Were appropriate methods used to account for censoring? | Answer **“Yes”** if censoring was handled using appropriate methods. Answer **“No”** if censoring occurred but was not addressed. Answer **“Unclear”** if censoring status is unclear. |
|  |  | In case of competing events, were appropriate methods used to account for them? | Answer **“Yes”** if competing events were reported and appropriately handled. Answer **“No”** if competing events occurred but were ignored. Answer **“NA”** if no competing events were relevant. Answer **“Unclear”** otherwise. |
| **Applicability** | 1.Participants | Were there concerns that the participants (and setting) did not match the research question? | Judge “**Low”**, **“High”**, or “**Unclear”** concern based on alignment with the target population and setting. |
|  | 2. Index Test | Were there concerns that the TMS-MEP assessment, its conduct, interpretation or threshold determination differed from the research question? | Judge concern based on whether the test conduct, interpretation, and thresholds reflect the intended clinical use. Respond with “Low” if variations in TMS equipment, test procedures, or interpretation likely had minimal impact on the accuracy of TMS-MEP results related to the research question. Answer “High” if the TMS-MEP test methods varied significantly from those outlined in the research questions and if there were specific concerns about their applicability. Otherwise, answer “Unclear”. |
|  | 3. Outcome | Were there concerns that the outcome(s) did not match the research question? | Evaluate the outcome measures based on their alignment with the research questions. Answer “Low” if the outcome measures matched the research questions, “High” if they did not (meaning there was a non-compatible choice of measures, and/or interpretation of results) and “Unclear” if there is insufficient information to make a definitive judgment. |
|  | 4. Flow and timing | Were there concerns that the time horizon did not match the research question? | Respond with “Low” if the time horizon aligns with the sensitive window for spontaneous neurological motor recovery following stroke based on the research questions. Answer “High” if the time horizon does not match with the research questions. Use “Unclear” if there is insufficient information to make a judgment. |

## Supplementary Material Section 3

### Assessment of technical items in prognostic single-pulse TMS-MEP studies

The included studies were evaluated using the checklist developed by Chipchase et al. (6), which assesses the reporting of technical and procedural elements specific to transcranial magnetic stimulation (TMS) in research involving single-pulse TMS-induced motor evoked potentials (TMS-MEPs) in the motor system following stroke.

This checklist was developed to standardize TMS testing procedures by ensuring that key technical and procedural elements are systematically reported. The original checklist comprises 31 items, including eight related to subject characteristics, 21 to methodological aspects, and two to analysis. For the purposes of the present review, the checklist was adapted to include only those items directly relevant to the evaluation of TMS-MEP studies employing a single-measure study design, defined as studies in which TMS-MEPs are assessed at a single time point without repeated-measures analyses.

Two independent reviewers (JS and MH) evaluated the included studies based on whether specific information was provided for each item. A third reviewer (GK) resolved any disagreements between the initial reviewers. Each item on the adapted checklist was categorized as 'Yes,' 'No,' or 'Not relevant' according to the criteria set forth by Osnabruegge et al (7), see Table 2. If a study reported that a specific procedure or item was performed, this was considered sufficient to classify the item as reported. Items were not categorized as 'controllable,' since most aspects of this area of TMS-MEP research are neither controlled for as covariates in statistical analysis nor balanced using questionnaires or normative data.

**Table 2.** Supportive rationales for the revised checklist evaluating the TMS-MEP-specific technical and procedural items in the included studies.

| Item | Rationale |
| --- | --- |
| Handedness of subjects. | Reporting handedness is essential when both left- and right-handed individuals are included, particularly in stroke populations. Cortical excitability measures such as resting motor thresholds (RMT) and MEP amplitude differ between dominant hemispheres and limbs, which may confound interpretation if unreported (8). Handedness supports appropriate comparison across subjects and studies and improves interpretability of laterality effects in TMS outcomes. |
| Subjects prescribed medication. | Central nervous system (CNS) active medications, including antidepressants, antipsychotics, anxiolytics, analgesics, and anticonvulsants can alter cortical excitability and thereby influence RMT and MEP amplitude. In stroke patients, certain medications may also modify seizure risk during TMS-MEP testing. Reporting prescribed medications is therefore critical to assess potential confounding effects, ensure safety transparency, and improve reproducibility. In this revised checklist, items related to CNS-active drugs and prescribed medication were combined to enhance clarity and reduce redundancy (9–12). |
| Position and contact of electromyography (EMG) electrodes. | Accurate reporting of surface EMG electrode placement, contact quality, and recording parameters (e.g., amplification, band-pass filtering) is necessary because these factors directly affect MEP detection and amplitude measurement. Insufficient details limits reproducibility and hampers comparison of outcome across studies using different EMG configurations (9). |
| Amount of relaxation/contraction of target muscles. | Cortical excitability and MEP characteristics are strongly influenced by the activation state of the target muscle. Reporting whether muscles are at rest or voluntarily contacted throughout testing is essential, as even low levels of background activity can substantially alter MEP amplitude and threshold (9,13,14). Clear documentation reduces within- and between-study variability and supports valid interpretation of TMS-MEP results. |
| Coil type (size and geometry). | Coil size and geometry determine the focality, depth, and spatial distribution of the induced magnetic field. Differences in coil design can lead to substantial variation in stimulated cortical volume and MEP characteristics (9). Reporting coil specifications is therefore necessary to enable methodological replication and meaningful comparison between studies |
| Direction of induced current in the brain. | The direction of the induced current influences which neuronal populations are activated during TMS. Coil orientation relative to the skull determines current direction and interacts with intracranial conductivity differences, which may be altered in stroke (9). Reporting current direction and pulse type (monophasic vs. biphasic) is essential for interpreting differences in neuronal recruitment, MEP size, and diagnostic or prognostic utility across studies. |
| Coil location and stability. | Precise documentation of coil positioning relative to anatomical landmarks or neuronavigation systems is required to ensure consistent cortical targeting. Information on how coil position is maintained (e.g., manual fixation, robotic assistance) is crucial, as small deviations can affect MEP amplitude (15,16). This reporting supports reproducibility and reduces measurement bias. |
| Type of stimulator used. | Different TMS stimulators vary in output characteristics, waveform generation, and maximum stimulation intensity. Reporting the manufacturer and model/version allows assessment of equipment-related variability and facilitates replication and cross-study comparison. |
| Stimulation intensity. | Stimulation intensity relative to RMT directly influences MEP amplitude and response probability. Because cortical excitability fluctuates over time and across individuals, explicit reporting of suprathreshold intensity parameters is essential for interpreting MEP outcomes and comparing results across protocols and populations (9,16,17). |
| Pulse shape. | Pulse shape (monophasic or biphasic) determines the temporal characteristics and directionality of the induced current, influencing neuronal recruitment and MEP expression. Reporting pulse shape and related parameters (e.g., peak-to-peak amplitude) is necessary to interpret differences in TMS effects and ensure methodological transparency (17). |
| Determination of the optimal hotspot. | The method used to identify the motor hotspot affects consistency and magnitude of elicited MEPs (18). Clear reporting of hotspot procedures supports reproducibility and reduces variability related to suboptimal or inconsistent cortical targeting. |
| Method for determining threshold. | Threshold determination procedures (e.g., criteria for defining RMT or active motor threshold, number of stimuli, MEP amplitude criteria) vary substantially across studies (19). Transparent reporting is essential to interpret stimulation intensity and compare thresholds across studies. |
| Number of TMS-MEP measures made. | The reliability of MEP measurements depends on the number of stimuli delivered. Evidence suggests that a minimum of five TMS stimuli is required to achieve acceptable within-session reliability in stroke populations (20). Reporting the number of TMS-MEP measures allows evaluation of data robustness and reliability. |
| Method for determining TMS-MEP size during analysis. | Explicit criteria for defining and quantifying MEPs (e.g., minimum amplitude, voltage threshold, peak-to-peak measurement) are necessary because analytical choices directly influence reported outcomes (9). Transparent reporting enhances comparability, reduces analytic bias, and supports reproducibility across studies. |

**Table 3.** The technical items related to the TMS-MEP testing from the 16 included studies using the revised Chipchase checklist.


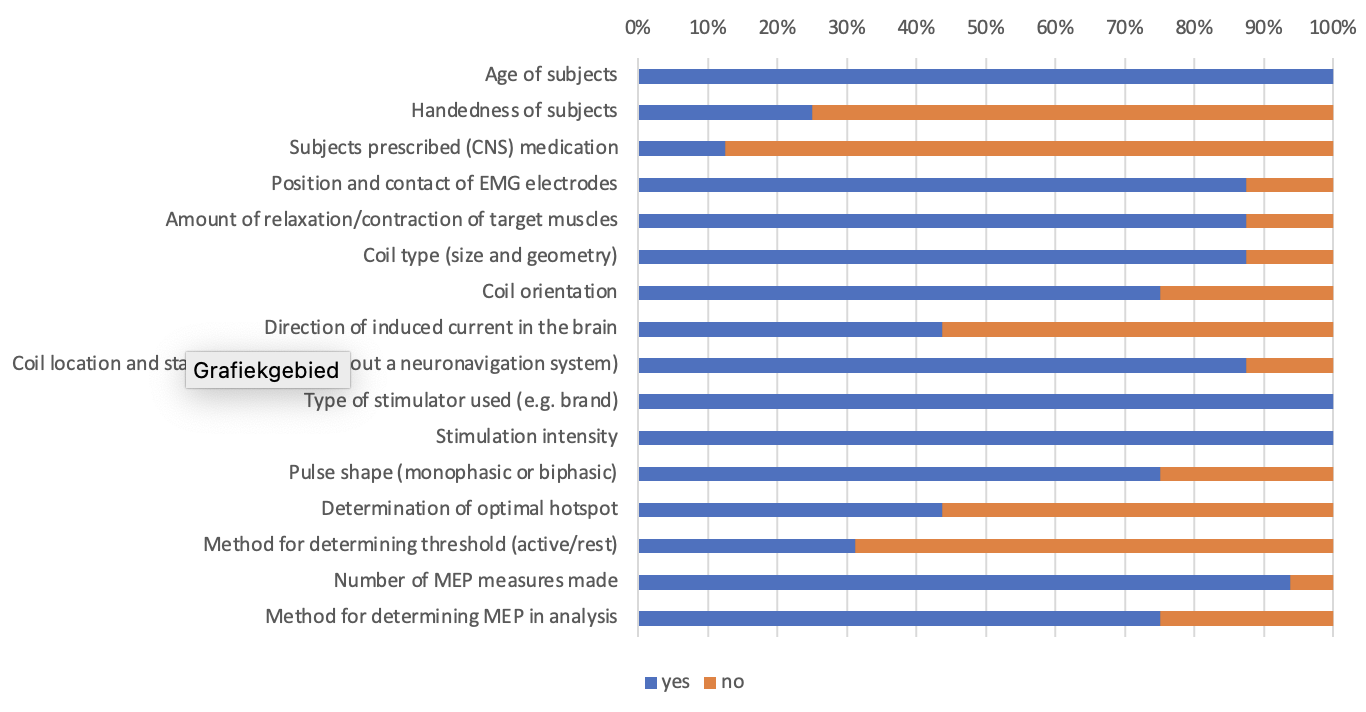


## References.

1. Malmivaara A. Applicability of evidence from randomized controlled trials and systematic reviews to clinical practice: A conceptual review. J Rehabil Med [Internet]. 2021;53(6):jrm00202. Available from: https://medicaljournalssweden.se/jrm/article/view/2392

2. Paulus W, Tergau F, Nitsche M, Rothwel J, Ziemann U, Hallet M. Proceedings of the 2nd International Transcranial Magnetic Stimulation and Transcranial Direct Current Stimulation Symposium, Gottingen, Germany, 11-14 June, 2003. Supplement to Clinical Neurophysiology Series. 1st ed. Vol. 56. New York: Elsevier Science & Technology; 2003. 3–452 p.

3. Rossini PM, Barker AT, Berardelli A, Caramia MD, Caruso G, Cracco RQ, et al. Non-invasive electrical and magnetic stimulation of the brain, spinal cord and roots: basic principles and procedures for routine clinical application. Report of an IFCN committee. Electroencephalogr Clin Neurophysiol. 1994 Aug;91(2):79–92.

4. Kwakkel G, Kollen B, Twisk J. Impact of time on improvement of outcome after stroke. Stroke [Internet]. 2006 Sep;37(9):2348–53. Available from: https://www.ahajournals.org/doi/10.1161/01.STR.0000238594.91938.1e

5. Cortes JC, Goldsmith J, Harran MD, Xu J, Kim N, Schambra HM, et al. A Short and Distinct Time Window for Recovery of Arm Motor Control Early After Stroke Revealed With a Global Measure of Trajectory Kinematics. Neurorehabil Neural Repair [Internet]. 2017 Jun 16;31(6):552–60. Available from: http://journals.sagepub.com/doi/10.1177/1545968317697034

6. Chipchase L, Schabrun S, Cohen L, Hodges P, Ridding M, Rothwell J, et al. A checklist for assessing the methodological quality of studies using transcranial magnetic stimulation to study the motor system: An international consensus study. Clinical Neurophysiology. 2012;123(9).

7. Osnabruegge M, Kanig C, Schwitzgebel F, Litschel K, Seiberl W, Mack W, et al. On the reliability of motor evoked potentials in hand muscles of healthy adults: a systematic review. Front Hum Neurosci [Internet]. 2023 Aug 31;17. Available from: https://www.frontiersin.org/articles/10.3389/fnhum.2023.1237712/full

8. Triggs WJ, Subramanium B, Rossi F. Hand preference and transcranial magnetic stimulation asymmetry of cortical motor representation. Brain Res [Internet]. 1999 Jul;835(2):324–9. Available from: https://linkinghub.elsevier.com/retrieve/pii/S0006899399016297

9. Groppa S, Oliviero A, Eisen A, Quartarone A, Cohen LG, Mall V, et al. A practical guide to diagnostic transcranial magnetic stimulation: Report of an IFCN committee. Clinical Neurophysiology [Internet]. 2012 May;123(5):858–82. Available from: https://linkinghub.elsevier.com/retrieve/pii/S1388245712000569

10. Ziemann U. TMS and drugs. Clinical Neurophysiology [Internet]. 2004 Aug;115(8):1717–29. Available from: https://linkinghub.elsevier.com/retrieve/pii/S1388245704001038

11. Rossi S, Hallett M, Rossini PM, Pascual-Leone A. Safety, ethical considerations, and application guidelines for the use of transcranial magnetic stimulation in clinical practice and research. Clinical Neurophysiology [Internet]. 2009 Dec;120(12):1–9. Available from: https://linkinghub.elsevier.com/retrieve/pii/S1388245709005197

12. Ziemann U, Reis J, Schwenkreis P, Rosanova M, Strafella A, Badawy R, et al. TMS and drugs revisited 2014. Clinical Neurophysiology [Internet]. 2015 Oct;126(10):1847–68. Available from: https://linkinghub.elsevier.com/retrieve/pii/S1388245714008372

13. Brown KE, Lohse KR, Mayer IMS, Strigaro G, Desikan M, Casula EP, et al. The reliability of commonly used electrophysiology measures. Brain Stimul [Internet]. 2017 Nov;10(6):1102–11. Available from: https://linkinghub.elsevier.com/retrieve/pii/S1935861X17308513

14. Edwardson MA, Avery DH, Fetz EE. Volitional muscle activity paired with transcranial magnetic stimulation increases corticospinal excitability. Front Neurosci [Internet]. 2015 Jan 12;8. Available from: http://journal.frontiersin.org/article/10.3389/fnins.2014.00442/abstract

15. Reijonen J, Säisänen L, Könönen M, Mohammadi A, Julkunen P. The effect of coil placement and orientation on the assessment of focal excitability in motor mapping with navigated transcranial magnetic stimulation. J Neurosci Methods [Internet]. 2020 Feb;331:108521. Available from: https://linkinghub.elsevier.com/retrieve/pii/S0165027019303784

16. Sondergaard RE, Martino D, Kiss ZHT, Condliffe EG. TMS Motor Mapping Methodology and Reliability: A Structured Review. Front Neurosci [Internet]. 2021 Aug 19;15. Available from: https://www.frontiersin.org/articles/10.3389/fnins.2021.709368/full

17. Pellegrini M, Zoghi M, Jaberzadeh S. The effect of transcranial magnetic stimulation test intensity on the amplitude, variability and reliability of motor evoked potentials. Brain Res. 2018;1700:190–8.

18. Rossini PM, Burke D, Chen R, Cohen LG, Daskalakis Z, Di Iorio R, et al. Non-invasive electrical and magnetic stimulation of the brain, spinal cord, roots and peripheral nerves: Basic principles and procedures for routine clinical and research application. An updated report from an I.F.C.N. Committee. Clinical Neurophysiology. 2015 Jun;126(6):1071–107.

19. Wassermann E. Variation in the response to transcranial magnetic brain stimulation in the general population. Clinical Neurophysiology. 2002;113(7).

20. Cavaleri R, Schabrun SM, Chipchase LS. The number of stimuli required to reliably assess corticomotor excitability and primary motor cortical representations using transcranial magnetic stimulation (TMS): a systematic review and meta-analysis. Syst Rev [Internet]. 2017 Dec 6;6(1):48. Available from: http://systematicreviewsjournal.biomedcentral.com/articles/10.1186/s13643-017-0440-8
